# Neutrophil Extracellular Traps Release following Hypoxic-Ischemic Brain Injury in Newborn Rats Treated with Therapeutic Hypothermia

**DOI:** 10.3390/ijms24043598

**Published:** 2023-02-10

**Authors:** Maria E. Bernis, Margit Zweyer, Elke Maes, Yvonne Schleehuber, Hemmen Sabir

**Affiliations:** 1 Department of Neonatology and Pediatric Intensive Care, Children’s Hospital, University of Bonn, 53127 Bonn, Germany; 2 Deutsche Zentrum für Neurodegenerative Erkrankungen (DZNE), 53127 Bonn, Germany

**Keywords:** neonatal, hypoxia, ischemia, hypothermia, inflammation, NETosis

## Abstract

The peripheral immune system plays a critical role in neuroinflammation of the central nervous system after an insult. Hypoxic-ischemic encephalopathy (HIE) induces a strong neuroinflammatory response in neonates, which is often associated with exacerbated outcomes. In adult models of ischemic stroke, neutrophils infiltrate injured brain tissue immediately after an ischemic insult and aggravate inflammation via various mechanisms, including neutrophil extracellular trap (NETs) formation. In this study, we used a neonatal model of experimental hypoxic-ischemic (HI) brain injury and demonstrated that circulating neutrophils were rapidly activated in neonatal blood. We observed an increased infiltration of neutrophils in the brain after exposure to HI. After treatment with either normothermia (NT) or therapeutic hypothermia (TH), we observed a significantly enhanced expression level of the NETosis marker Citrullinated H3 (Cit-H3), which was significantly more pronounced in animals treated with TH than in those treated with NT. NETs and NLR family pyrin domain containing 3 (NLRP-3) inflammasome assembly are closely linked in adult models of ischemic brain injury. In this study, we observed an increase in the activation of the NLRP-3 inflammasome at the time points analyzed, particularly immediately after TH, when we observed a significant increase in NETs structures in the brain. Together, these results suggest the important pathological functions of early arriving neutrophils and NETosis following neonatal HI, particularly after TH treatment, which is a promising starting point for the development of potential new therapeutic targets for neonatal HIE.

## 1. Introduction

Neonatal hypoxic-ischemic encephalopathy (HIE) is a leading cause of neonatal mortality and is associated with a variety of life-long morbidities [1,2,3,4,5]. The incidence of HIE ranges from 1–8/1000 live births in developed countries to as high as 26/1000 live births in developing countries [4,6,7]. Currently, the standard therapeutic treatment for neonatal HIE is therapeutic hypothermia (TH) [4,5]. TH reduces the risk of death and the possibility of adverse long-term neurodevelopmental outcomes [4,5]. TH can reduce primary injury and prevent secondary injury to the brain following HI [8]. TH influences multiple aspects of brain physiology in the acute, subacute, and chronic stages of HI [9]. However, TH is only partially neuroprotective in some cases, and is not neuroprotective in severe cases of HIE or in cases of neonatal infection combined with HIE [10,11,12].

Sterile inflammation has been strongly associated with acute conditions such as stroke, traumatic brain injury, and Alzheimer’s disease, which involves the infiltration of peripheral leukocytes, particularly neutrophil cells, into the damaged brain [13,14,15,16,17,18,19,20]. Unlike neutrophil cells in adult tissues, neonatal neutrophils are believed to be immature and show less extravasation to the injured brain, leading to the assumption that neutrophils are clinically less relevant [21,22]. However, recent evidence has shown that neutrophils can penetrate the blood–brain barrier and accumulate in the parenchyma of the injured neonatal brain [23,24]. Most studies in neonatal cases rely on the analysis of peripheral blood as a routine evaluation [25,26,27] or by ex vivo stimulation in culture [28]. However, the exact role of peripheral neutrophils and infiltrated neutrophils in the brain after hypoxia-ischemia (HI) under sterile inflammation is unknown, and even less is known about the effect of TH on neutrophil function [23,29,30].

In cases of neonatal HIE, little evidence has shown that the recruitment of peripheral and cerebral immune cells into damaged brain areas play a crucial role in HI brain injury [31,32,33,34,35,36,37,38,39], and the level of neutrophil infiltration following HI is associated with an increase in adverse outcomes [26,27,39,40,41]. 

Early neutrophils infiltrating the injured area are mostly phagocytic cells. However, neutrophil extracellular trap (NETs) formation functions as an alternative defense mechanism once the maximum phagocytic activity is reached [15,42]. NETs are composed of webs of extracellular DNA decorated with histones (Cit-H3), myeloperoxidase (MPO), and elastase (NE), which contribute to pathogen clearance in a process called NETosis [43,44]. In contrast, excessive NETosis formation promotes inflammation and tissue damage [18].

The nucleotide-binding domain, leucine-rich repeat protein (NLRP-3) inflammasome has been involved in neonatal brain injury [45,46]. NETs and the NLRP-3 inflammasome are closely linked to the development of several diseases in adult humans [47,48]. A recent publication in a subarachnoid hemorrhage brain injury model showed that NLRP-3 inflammasome assembly in neutrophil cells is regulated by peptidyl-arginine deiminase 4 (PAD4) and caspase-1 cleavage and promotes NETosis formation under sterile conditions [49]. However, little is known about NLRP-3 and the role in neutrophil function in neonatal HIE [49,50,51].

Based on these preliminary data, we aimed to demonstrate a link between neonatal HI brain injury, NETosis formation, and the influence of TH treatment on NETosis formation. In addition, we aimed to demonstrate the potential role of the NLRP-3 inflammasome in our model.

## 2. Results

### 2.1. Neonatal HI Causes an Increase in Circulating Peripheral Neutrophils in the Blood with an Increase in Infiltrating Neutrophils in the Brain Dependent on Treatment

**Figure 1:**
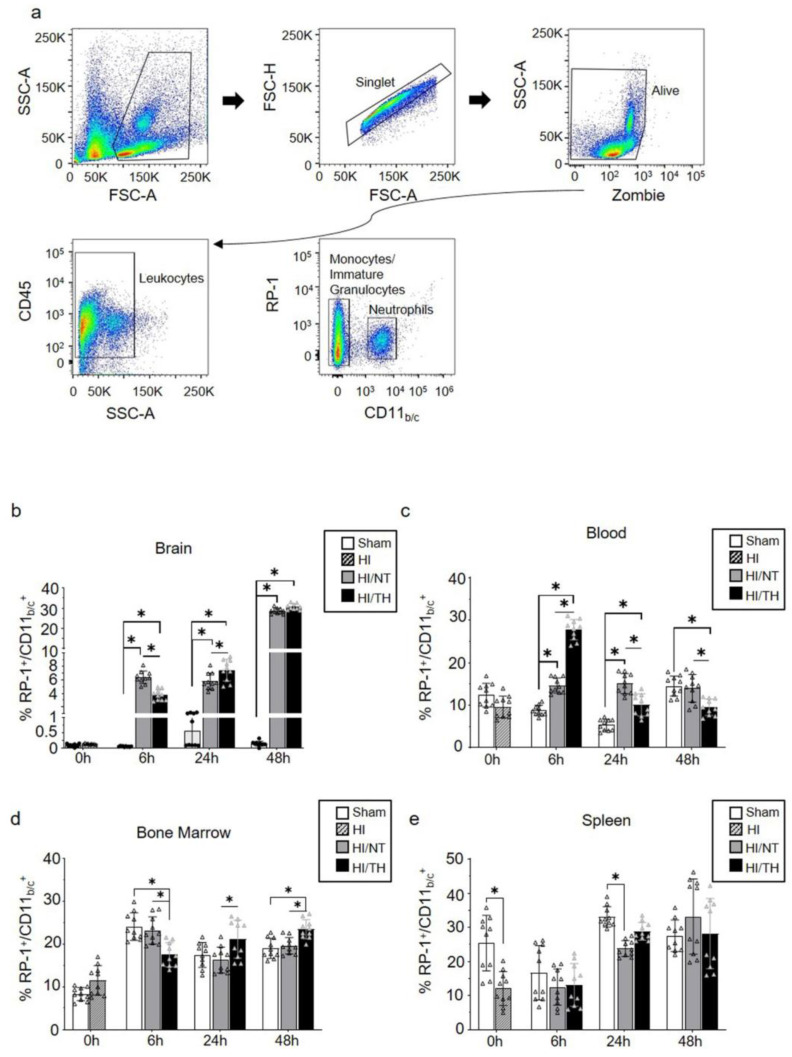
Circulating neutrophils over time after HI following NT and TH treatment in different samples. The proportion of neutrophils was quantified by flow cytometry using specific antibodies against the rat tissues. Gate strategy (**a**) was performed considering granulocyte, single, and live cells, following all CD45 positive cells, and considering the percentage of all positive RP-1 respect CD11_b/c_ cells. The percentage of all RP-1^+^/CD11_b/c_^+^ neutrophils in the brain (**b**), blood (**c**), bone marrow (**d**), and spleen (**e**) at the indicated time points after HI following NT or TH compared to the sham sample. Sham (white column), HI (grey with cross-line column), HI/NT (grey column), and HI/TH (black column). Data of ten biological replicates (n = 10) per time point and condition (see Section 4). Two-way ANOVA was used with a * *p* < 0.05. Data are expressed as mean ± SD.

Currently, the temporal expression of circulating neutrophils in the periphery and brain after neonatal HI, particularly after TH treatment, is not well characterized. To characterize the proportion of neutrophils in our experimental model after different treatments and time points, we performed flow cytometry on the leukocytes isolated from the brain, blood, bone marrow, and spleen. Using a gate strategy in which all CD45^+^ cells were included, we took advantage of the higher granularity of neutrophils and counted the percentage of cells RP-1^+^ with respect to the percentage of CD11_b/c_^+^ cells (Figure 1a). We observed a significant increase in isolated neutrophils from the ipsilateral brains 6 and 48 h after HI, compared with the sham group for both treatment groups (Figure 1b). The percentage of neutrophils in the sham group did not show any significant changes over time in brain samples (Figure 1b). Six hours after HI, we observed a significant increase in the NT group compared with the TH group (Figure 1b), whereas 24 h after HI, the TH group showed a significant increase compared to the NT group (Figure 1b). No significant differences were observed 48 h after HI between the two treatments (Figure 1b). Neutrophils in blood samples showed a significant increase at 6 h and 24 h after HI in both treatments when compared to the sham group, similar to what we observed in the brain at the corresponding time points analyzed (Figure 1c). Six hours after HI, TH showed a significant increase compared to NT, whereas 24 h after HI, TH showed a significant decrease compared to NT. Forty-eight hours after HI, a significant decrease was observed in the TH group compared to that in the sham and NT groups. In contrast to the observation of neutrophils in blood and brain samples, the bone marrow TH group samples showed a significant decrease at 6 h after HI when compared to the sham and NT groups (Figure 1d), while at 24 h and 48 h after HI, TH showed a significant increase in the NT group. Spleen samples showed a significant decrease immediately after HI in the HI group compared to the sham group (Figure 1e). Twenty-four hours after HI, only the NT group showed a significant decrease compared to the sham group. No significant changes were observed at the other time points between the treatments.

### 2.2. Temporal Induction of NETosis Is More Pronounced in the Injured Brain after TH

**Figure 2:**
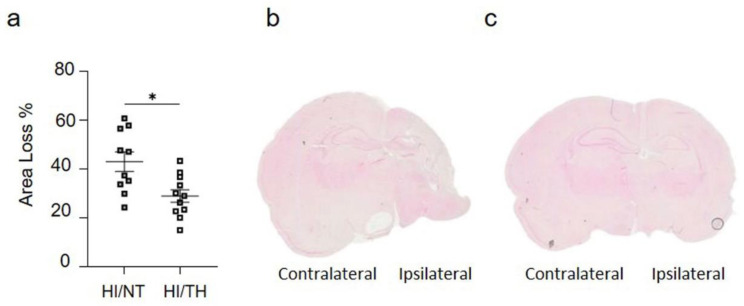
(**a**) Hemispheric area loss (%). P7 rat pups underwent ligation of the left carotid artery followed by 90 min of hypoxia. Pups were randomized to either 5 h of NT (37 °C) or 5 h of TH (32 °C) after hypoxia and were sacrificed after 7 days of survival. Therapeutic hypothermia significantly reduced brain area loss. (**b**,**c**) Representative image of coronal section from NT brain (**b**) and TH brain (**c**) staining with eosin showing the damage over the ipsilateral side on the NT treatment compared to TH treatment. n = 21 animals (n = 10 NT and n = 11 for the TH group). Student’s *t*-test was performed with a * *p* < 0.05. Data are expressed as median (interquartile range).

**Figure 3:**
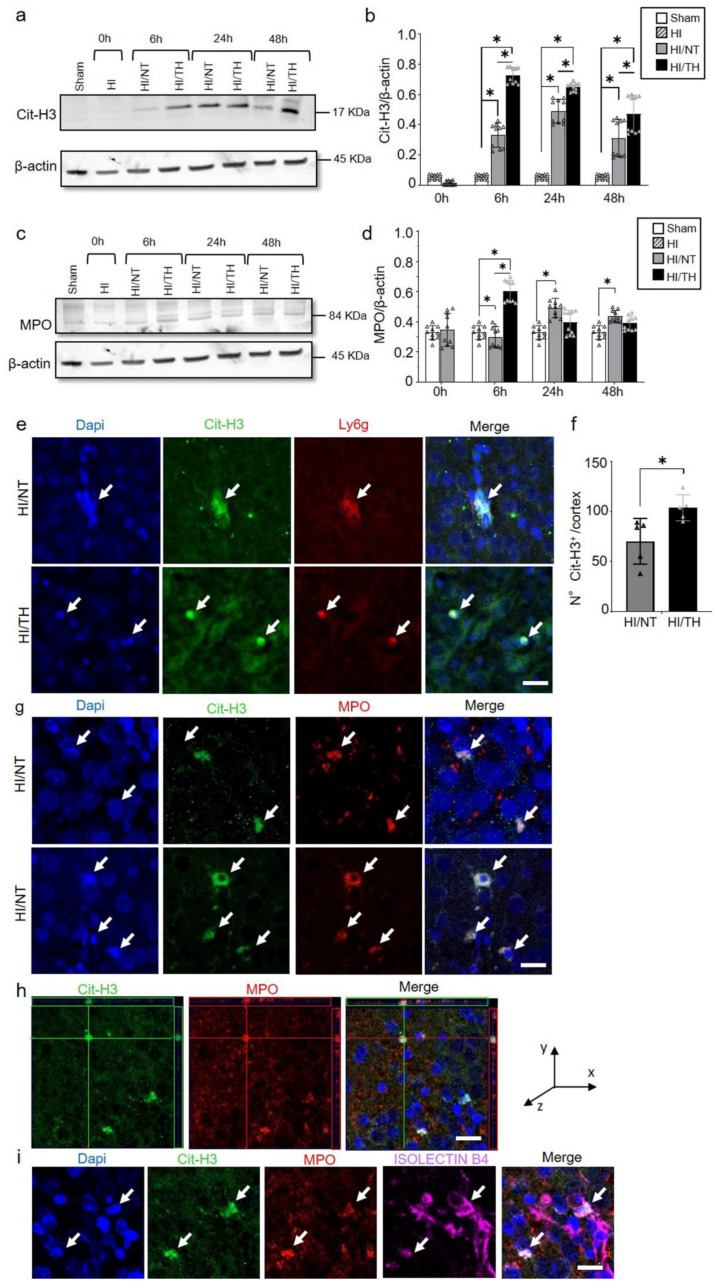
Temporal expression of NETosis in the ipsilateral brain samples after HI-NT or HI-TH. Representative Western blotting images for all time points and conditions (**a**) for neutrophil NETosis marker Cit-H3 and β-actin as loading control, or (**c**) MPO marker and β-actin. (**b**) Quantitative analysis of Cit-H3 expression or (**d**) MPO expression in neutrophils isolated from the brain samples at the indicated time points after HI following NT or TH. The optical density was measured, and the ratio against the loading control β-actin was calculated. Sham (white column), HI (grey with cross-line column), HI/NT (grey column), and HI/TH (black column). Data of ten biological replicates (n = 10) per time point and condition (see Section 4). Two-way ANOVA was used for c and e while Student’s *t*-test was used for g with a * *p* < 0.05. Data are expressed as the mean ± SD. (**e**) Representative images of positive NETosis in the cortical area from the ipsilateral side of the brain 24 h post-HI. The following antibodies were used: Cit-H3 (green), neutrophils marker Ly6g (red), and nuclear marker Dapi (blue). The white arrow indicates positive Cit-H3/Ly6g NETs and the corresponding nuclear staining. Images were taken with confocal LSM900, with a 20× objective and a zoom of 3×. Scale bar = 20 µm. (**f**) Quantitative analyses of the cortical area showing the number of positive Cit-H3 cells in the NT group (grey column) compared to the TH group (black column) (24 h after HI; data from five biological replicates (n = 5)). Student’s *t*-test was used with a * *p* < 0.05. Data are expressed as the mean ± SD. (**g**) Representative image of double-immunostaining for NETs and the neutrophil marker MPO in the cortical area from the ipsilateral side of the brain 24 h post-HI. The following antibodies were used: Cit-H3 (green), neutrophils marker MPO (red), and the nuclear marker Dapi (blue). The white arrow indicates positive Cit-H3 NETosis and the corresponding MPO-positive cells. Images were taken with confocal LSM900, with a 20× objective and a zoom of 2×. Scale bar = 10 µm. (**h**) Representative three-dimensional pictures of double immunostaining for Cit-H3 and MPO in the cortical area. Each square on top of each picture represents a dimensional plane (up—y/x, right side y/z) where it is possible to observe the co-localization between both markers. Scale bar = 20 µm. (**i**) Representative images of multiple immunostaining for NET marker Cit-H3 (green), neutrophil marker MPO (red), blood vessel marker Isolectin 4 (purple), and nuclear marker Dapi (blue) in the cortex area from the ipsilateral side of the brain 24 h after HI. The white arrow indicates positive Cit-H3 NETosis and the corresponding MPO-positive cell. Images were taken with confocal LSM900, with a 20× objective and a zoom of 2×. Scale bar = 10 µm.

As demonstrated previously, TH was neuroprotective after HI in our model and reduced the area of injury (Figure 2a–c) [10,52,53]. However, the immune response of peripheral cells in the injured brain, particularly after TH, remains unknown. Several studies have reported the presence of neutrophil NETosis in noninfectious environments, such as stroke injury [15,43]. Nevertheless, the presence of NETs in the brains of neonates following HI remains unknown, and even less is known about NETs formation after TH treatment. Here, we demonstrate the presence of NETosis in brains undergoing neonatal HI following treatment with either NT or TH. Levels of Cit-H3 (a common NETs marker) were examined in isolated neutrophil samples from the ipsilateral side of the brain at different time points using Western blotting. We observed a significant increase in the levels of Cit-H3 from 6 to 48 h after HI, regardless of the treatment, compared to the sham group (Figure 3a,b). Interestingly, we observed at those time points a significant increase in the level of Cit-H3 in the TH group compared with that in the NT group (Figure 3a,b). No significant changes were observed in the samples immediately after HI (Figure 3a,b). The sham group did not show any significant changes over time. We also analyzed the expression level of MPO (Figure 3c,d), a common protein found in NETs and a common neutrophil marker, in isolated neutrophil samples from the ipsilateral side of the brain at different time points, following treatment with NT or TH. Six hours after HI, a significant increase was observed in the TH group compared to the sham and NT groups (Figure 3c,d). However, 24 and 48 h after HI, a significant increase was observed in the NT group compared to the sham group, while MPO expression after TH at those time points showed a slight increase, but was not significant when compared to the sham group (Figure 3c,d). Western blotting of blood samples did not show any Cit-H3-positive immunostaining (Supplementary Figure S1). Immunostaining of the cortical area of the ipsilateral side of the brain 24 h after HI (Figure 3e, white arrows) showed Cit-H3-positive staining co-localizing with Ly6g (a common neutrophil marker) and MPO (Figure 3g) in the NT, as well as in TH-treated brains. A three-dimensional orthogonal picture showed the co-localization of Cit-H3 and MPO in the cortical area, indicating that our Cit-H3-positive marker was co-localized with the MPO-positive marker (Figure 3h). We did not observe positive Cit-H3 staining in the sham group. Quantitative analysis showed a significant increase in the number of positive NETs in the cortex in the TH group compared to that in the NT group (Figure 3f) 24 h after HI, as previously observed (Figure 3a,b). We observed typical cloudy and multi-lobular nucleus staining, indicating neutrophils under NETosis accumulation (Figure 3e,g, HI/TH treatment). We also observed neutrophils in the third ventricle (Figure 3i) with co-localization of Cit-H3 and MPO in areas enriched with the blood vessel marker isolectin-4 (Figure 3i, purple).

### 2.3. Temporal NETosis and Interaction with Other Cells in the Injured Brain after HI following TH Treatment

**Figure 4:**
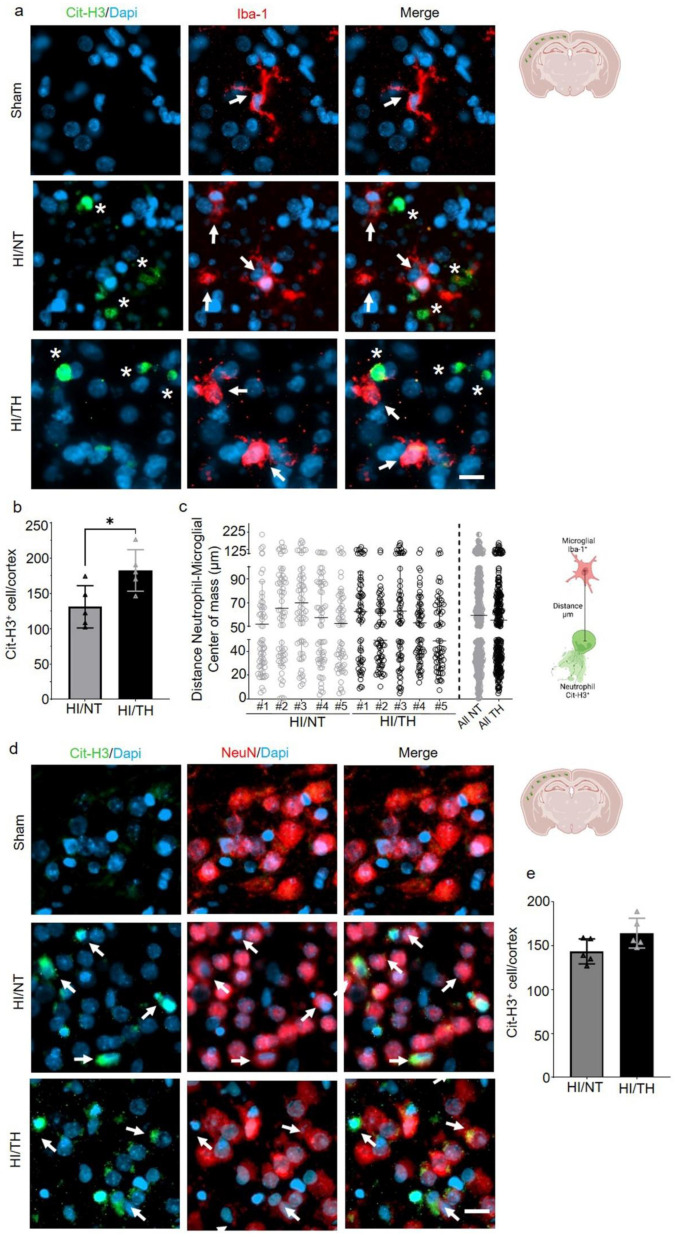
NETosis and its interactions with other cells in the brain after HI injury. Immunostaining was performed on brain slices at 24 h post-HI and representative images were taken from the cortex on the ipsilateral side of the brain. (**a**) Positive Cit-H3 cells in green show that the formation of NETosis was near positive microglial cells (red) in both NT and TH treatment groups. White arrows indicate positive Iba-1 microglial cells, while white asterisks indicate positive Cit-H3 NETs. Clear amoeboid and activated microglial cells were observed in the NT and TH groups, while ramified and resting microglia cells were observed in the sham group. The nuclear marker Dapi is shown in blue. (**b**) Quantitative analyses of the cortical area showing the number of positive Cit-H3 cells in the NT group (grey column) compared to the TH group (black column) (24 h after HI; data from five biological replicates (n = 5)). Student’s *t*-test was used with a * *p* < 0.05. Data are expressed as the mean ± SD. (**c**) Quantitative analyses 24 h after HI of the distance between the center of mass between neutrophil and microglial cells. #1–5 represent different animals. n = 5 with a total of 60 cells in the cortical areas per animal counted. Data are expressed as the mean ± SD. (**d**) Positive Cit-H3 cells (green) are proximal to positive NeuN cells (red) regardless of the treatment used: NT or TH. The nuclear marker Dapi is shown in blue. (**e**) Quantitative analyses of the cortical area showing the number of positive Cit-H3 cells in the NT group (grey column) compared to the TH group (black column) (24 h after HI; data from five biological replicates (n = 5)). Data are expressed as the mean ± SD. Images were taken with Axioscan microscope with an objective of 20×. Scale bar = 10 µm.

Having confirmed the presence of NETosis in the brain after HI followed by NT or TH, we next investigated the interaction between NETosis and microglia cells. It has been demonstrated that microglia can induce the formation of NETs, called MiETs (Microglia Extracellular Traps), after an insult in the brain or in some neurodegenerative diseases [54]. By performing a double immunohistochemistry staining with the microglia marker, calcium-binding adaptor molecule 1 (Iba1) and Cit-H3, we observed microglia in the proximity of Cit-H3-positive cells 24 h after HI. However, we could not observe co-localization between neutrophils under NETosis or microglia cells in the cortical area of the brain under NT, nor following TH, indicating that Cit-H3-positive cells are from neutrophils (Figure 3a, white asterisk—NETs and white arrows—microglia). We observed that all microglial cells in the NT and TH groups presented an amoeboid shape, typically in activated microglia cells. The sham group did not show any positive immunostaining for Cit-H3 and showed positive Iba-1 microglia cells with a typical ramified shape similar to resting microglia (Figure 4a, white arrows). Quantitative analysis showed a significant increase in the number of positive NETs in the cortex in the TH group compared to that in the NT group (Figure 4b) 24 h after HI, as previously observed (Figure 3a,b). In order to measure possible interaction between neutrophil and microglial cells, we measured the distance between both cell types’ center of mass. The analysis showed that neutrophil and microglia cells’ average distance is approximately 50 µm, showing at the time analyzed that both cells were not interacting in both treatments, NT and TH (Figure 4c). We also analyzed the interaction between neurons and NETosis (Figure 4d, white arrows). For this purpose, we performed immunostaining with Cit-H3 in combination with the neuronal marker NeuN. At the time point analyzed, we observed positive immunostaining for neutrophils under NETosis, and positive NeuN cells, but no difference was observed between the two treatments (Figure 4d). Quantitative analysis showed a slight but not significant increase in the number of positive NETs in the cortex in the TH group compared to that in the NT group (Figure 4e) 24 h after HI, as previously observed (Figure 3a,b). We did not observe positive Cit-H3 in the sham group (Figure 4a,d).

### 2.4. Early Increase in NLRP-3 Inflammasome Corresponds with an Early Increase in NETosis following Therapeutic Hypothermia

**Figure 5:**
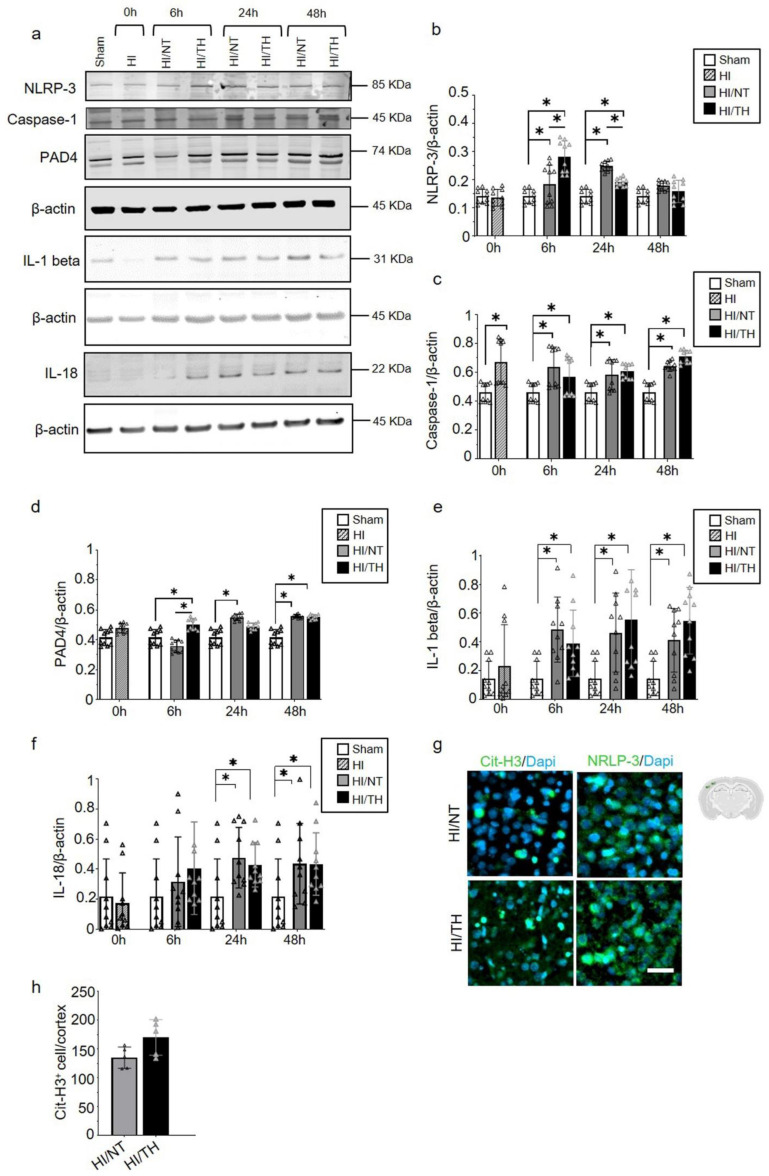
Temporal induction of NETosis increased the expression of NLRP-3 inflammasome in ipsilateral brain samples after HI-NT or HI-TH treatment. Representative Western blotting images for all time points and conditions (**a**) for the NLRP-3 inflammasome marker, Caspase-1, IL-1beta, IL-18, and PAD4, and loading control β-actin. (**b**) Quantitative analysis of NLRP-3 inflammasome, (**c**) Caspase-1, (**d**) IL-1beta, (**e**) IL-18, and (**f**) PAD4 expression in neutrophils isolated from brain samples at the indicated time points after HI following NT or TH. Optical density was measured, and the ratio against the loading control β-actin was calculated. Data of ten biological replicates (n = 10) per time point and condition (see Section 4). Two-way ANOVA was used with a * *p* < 0.05. Data are expressed as the mean ± SD. (**g**) Representative images of positive NETosis in the cortical area from the ipsilateral side of the brain 24 h after HI in areas with strong NLRP-3-positive staining. The nuclear marker Dapi, in blue. Scale bar = 10 µm. Images were taken with Axioscan microscope, with an objective of 20×. (**h**) Quantitative analyses of the cortical area showing the number of positive Cit-H3 cells in the NT group compared to the TH group (24 h after HI; data from five biological replicates (n = 5)). Data are expressed as the mean ± SD. (**b**–**f**,**h**): sham (white column), HI (grey with cross-line column), HI/NT (grey column), and HI/TH (black column).

While the role of the NLRP-3 inflammasome in non-sterile NETosis formation has been described in other models, such as adult stroke [51,55,56], little is known about sterile inflammatory conditions in neonatal HI. Using isolated neutrophil cell samples from brain tissue, we observed a significant increase in NLRP-3 expression in neutrophils isolated from the ipsilateral side of the brain at different time points (Figure 5a,b). Our data showed a significant and gradual increase from 6 h to 24 h after HI, which declined 48 h after HI in the NT group compared to the sham group (Figure 5a,b). The TH group showed a significant increase 6 h after HI and a significant decline at 24 h after HI compared to the sham group. Forty-eight hours after HI, the values remained comparable to those of the sham group (Figure 5a,b). Interestingly, 6 h after HI, the TH group showed a significant increase compared to the NT group, whereas 24 h after HI, the opposite effect was observed (Figure 5a,b). We analyzed the levels of Caspase-1 as an important factor in NLRP-3 inflammasome formation, and we observed a significant increase at all-time points regardless of the treatment when compared to the sham group (Figure 5a,c). Our analysis of IL-1beta and IL-18 showed a significant increase in both groups at 6h after HI (Figure 5a,d,e). We observed a significant increase in IL-1 beta in both the NT and TH groups from 6 h, which remained high throughout the times analyzed compared to the sham group (Figure 5a,e). A slight increase at 24 h was observed for the TH group when we compared it to the NT group. Similar results were observed for IL-18, with a significant and gradual increase in IL-18 from 6 h to 24 h after HI for both groups, which remained constant at 48 h compared to the sham group (Figure 5a,f). The TH group showed a slight increase 6 h after HI compared with the NT group (Figure 5a,e). It was previously demonstrated that PAD4 plays a crucial role in NETosis formation [57]. Six hours after HI, the TH group showed a significant increase compared with the sham and NT groups (Figure 5a,f). However, 6 h after HI, only the NT group showed a significant increase compared with the sham group (Figure 5f). A slight increase, but not statistically significant, was observed in the TH group 24 h after HI compared to that in the sham group. Forty-eight hours after HI, both treatments showed a significant increase compared with the sham group (Figure 5f). These changes in the expression level of PAD4 corresponded to those observed in NLRP-3, 6 h and 24 h after HI for both treatments (Figure 5a,b). These results corroborate previous evidence showing that NLRP-3 inflammasome assembly in neutrophils is supported by PAD4 and may promote NETosis formation [49]. We performed immunostaining using brain slices from 6 h after HI for both treatments, and our results showed that in the cortical area, an increase in positive Cit-H3 cells corresponded to areas with an increase in NLRP-3-positive cells (Figure 5g). Quantitative analysis showed a slight but not significant increase in the number of positive NETs in the cortex in the TH group compared to that in the NT group (Figure 5h) 24 h after HI, as previously observed (Figure 3a,b).

## 3. Discussion

Here, we demonstrated that after HI, neutrophils infiltrate the brain following NT or TH treatment. We observed an increase in the number of peripheral neutrophils over time, particularly in those circulating in the blood, where we observed that TH increased the number of neutrophils immediately after TH treatment, whereas the NT response was constant over the time analyzed. Interestingly, the number of infiltrated neutrophils in the brain increased over time. In particular, TH showed a time-dependent increase compared with the NT group. In a neonatal HI model, we demonstrated the presence of NETosis, particularly following treatment with TH. NETs were highly present in the brains of those treated with TH at all-time points, particularly early after TH treatment followed by a subsequent decrease 48 h post-HI, while in samples treated with NT, the levels of NETs showed a period of increment 24 h after HI, with a subsequent decrease over time. We also found that NETs co-localized with a specific neutrophil marker (Ly6g) as well as MPO, another protein found in NETs, corroborating that they belong to neutrophil cells. We demonstrated that the NLRP-3 inflammasome was highly expressed at the same time points as we observed with the expression levels of NETosis after TH. Our results show a possible mechanism for NETosis formation in the neonatal HI brain and a possible link to the underlying mechanism of TH by promoting the formation of NETs in the injured brain. These results open the door for new research to address the role of NETosis and HI following TH, particularly in cases in which TH is not beneficial.

Neonatal HI is one of the primary causes of acute neonatal brain injury, leading to a high mortality rate and long-term neurological disabilities [3]. Currently, TH is the standard treatment; however, it is not beneficial in severe HI cases [10,58,59,60]. Hypothermia-induced neuroprotection may be due to decreased metabolism, reduced generation of radicals, ameliorated inflammation, and inhibition of excitotoxicity and apoptosis [9]. In addition, it plays an important role in regeneration and in the mediation of structural plasticity by the induction of cold-shock proteins, such as the RNA-binding protein RBM3 [8,61,62]. Inflammation plays a critical role in neonatal HI and is a major contributor to the pathophysiology of neonatal brain injury [3,38,39,63,64]. Neutrophils are the most abundant leukocytes and the first immune cell type from the periphery that is recruited to the site of tissue damage [19,20,23,65,66]. The neutrophil response to an injury in the brain is mainly due to the release of signals from immune cells, such as microglia [38,67,68], following infiltration in the brain by blood–brain barrier disruption [19,57]. It has been demonstrated in adult models of stroke that neutrophils do not cross the perivascular space in the brain in a substantial number after transient ischemia and are more motile than the parenchyma area [69,70]. This represents an important limitation in the isolation of infiltrated neutrophils from the brain.

Studies in human patients with stroke have shown a high number of peripheral neutrophils being associated with the severity of injury [71], as well as in neonatal HI associated with poorer neurological outcomes [26]. It has been shown that TH has a minimal effect on peripheral immune cell counts in neonatal HI cases [72]; however, HI neonates undergoing TH have altered inflammatory responses compared to healthy infants [73]. It is commonly believed that an increase in neutrophil count after HIE is possibly associated with an infection and adverse outcome; however, evidence has shown no significant changes in the neutrophil count in asphyxiated newborns [27], even less during TH [72]. Recently, studies in adult animal models of stroke have demonstrated the critical role of neutrophils in the development of inflammation [17,18,19,20,57,65]; however, few studies using neonatal HI models have supported this, and the exact role of neutrophils is poorly understood [23,65]. Interestingly, in both cases, infiltration of neutrophils was observed at early time points after an insult, implying a short survival time for these leukocytes [44,74,75].

While all these studies are of absolute importance for the prediction of outcome, their function in the brain is still a matter of debate, and it is difficult to assess clinically because of the immaturity of the immune system in neonates compared to adults.

Neutrophils employ several strategies to modulate inflammation, including formation of neutrophil extracellular traps (NET) and posterior NETosis [44,74,75]. Previously, NETosis was commonly associated with infection, but recently it was associated with a response to sterile conditions [17,18,57,76,77]. NETosis is the result of projections of decondensed chromatin and granular contents, such as MPO, Elastase, and Cit-H3, which effectively immobilize pathogens or cell debris [74]. However, recent evidence has shown that peripheral NETs can have fewer typical markers, such as MPO, even when Cit-H3 is elevated, mainly because of a change in the surface that can affect the binding of MPO to NETs in the presence of histones [78,79].

Neutrophils play a beneficial role after activation, however, their improper function can lead to tissue damage or even exaggerate an inflammatory response [80]. However, how neutrophils regulate their inflammatory response, or which role NETosis plays, remains an area of thrilling but unresolved research [16,81]. One possible role of neutrophils under sterile conditions may involve autophagy of cell death and cell debris [82]. Because not all neutrophils trigger NETs, they may have different functions [83,84,85]. However, there is a lack of consensus regarding neutrophil subtypes, and even less consensus about the types of NETs.

Induction of citrullinated histone-3 (Cit-H3) has been associated with adverse outcomes in adult animal models of cerebral ischemia and intracerebral hemorrhage [17,18,77]. NET-like structures are linked to amplified neurotoxicity in the brain after IL-1β-induced transendothelial migration of neutrophils [86]. Similarly, CitH3-positive neutrophils were found in perivascular spaces, in the brain parenchyma near blood vessels, and in the lumen of the capillaries in an animal model of permanent middle cerebral artery occlusion (MCAO) [20]. In a mouse model of Alzheimer’s disease, NETosis was detected in areas containing amyloid-β (Aβ) deposits [76]. In an adult model of MCAO, it has been shown to induce NETosis in the post-ischemic brain, and suppression of NET formation mitigates delayed inflammation and vessel damage [17,18,57]. Therefore, a growing body of evidence implicates NETosis in the inflammatory response in noninfectious CNS diseases [87]. However, information regarding the role of NETosis in sterile brain inflammation in neonate HI cases is lacking.

The therapeutic potential of induced neutropenia and/or NETosis inhibition in adult animal models of stroke [17,18,77] showed delayed immune cell infiltration and mitigated damage to blood vessels. However, a reduction in infarct volume was not detected in most brains. Strategies to reduce these NETs or even enhance their function are important therapeutic targets, and this has become the next goal to be addressed in our laboratory. Most commercially available drugs target NETs formation but do not specifically target NETs formation pathways [42,88,89,90]. Therefore, specific drugs targeting NETs’ components may serve as potential therapeutic targets. NETs could also function as a potential biomarker, not only to measure the degree of injury, but also to provide information about the effect of certain treatments, considering the changes in different proteins that form NETs. However, the techniques for determining NETs are not trivial, and, unfortunately, quantitative changes in NETs formation may not be directly associated with the progression or amelioration of an injury.

Early activation of the NLRP-3 inflammasome has been previously shown in a model of traumatic brain injury, where the authors described the neuroprotective effect of TH by dysregulating the inflammasome activation [91]. However, while the exact role of TH in the NLRP-3 inflammasome has been demonstrated in adult animal models [92,93], currently there is little evidence of the role of TH on the NLRP-3 inflammasome in the pathogenesis of neonate HI [93]. We previously demonstrated that the NLRP-3 inflammasome is activated in microglial cells in our LPS pre-sensitized neonatal HI model after HI [46,94]. NLRP-3 has also been implicated in various sterile inflammatory diseases [55,95]. The role of the NLRP-3 inflammasome in neutrophil activation is still debated [49,50,96]. We observed the activation of the NLRP-3 inflammasome in purified neutrophil samples from the ipsilateral side of the injured brain at different time points after HI. When we compared NT and TH, we observed an opposite effect on the activation of the NLRP-3 inflammasome. Immediately after treatment, TH showed an increase in NLRP-3 inflammasome expression compared to NT; at 24 h after hypoxia, the effect was the opposite. Notably, immediately after treatment, we observed an increase in the expression of Cit-H3 in the TH group compared to that in the NT group. We also observed a progressive increase in caspase-1 (another important factor in inflammasome activation) [51] and PAD4 over time [49]. This observation over time shows a temporal activation, especially in the first hours after HI, as has been seen previously in our laboratory in microglial cells [46]. These preliminary data showed a possible role of the NLRP-3 inflammasome, not only in regulating the inflammatory response of microglial cells, but also from neutrophils during the inflammatory resolution after HI brain injury. However, more studies are needed in this field to shed light on this inflammatory complex mechanism and its role in the activation of immune cells in the brain after HI, using specific inhibitors or transgenic mouse models.

A clear understanding of the pathological function of early infiltrating neutrophils in the neonatal HI brain and its subsequent NETosis formation is crucial not only for understanding the pathophysiology of neonatal HI but also to bring some light to our limited knowledge about the mechanism of action of TH. This could be a starting point for the development of further research areas in the topic, and to improve our understanding of the mechanism of action behind TH, and how this could lead to the development of novel therapies that could further reduce mortality and improve long-term neurodevelopmental outcomes of asphyxiated newborns.

In conclusion, our results open the door for a wide range of studies in the neonatal HIE field, particularly in understanding the mechanism behind TH and how peripheral immune cells might play a relevant role in the neuroprotective mechanism depending on the severity of the insult.

## 4. Material and Methods

### 4.1. Animals and Experimental Procedure

**Figure 6:**
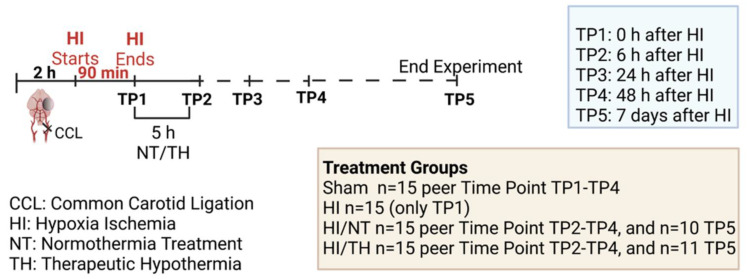
Experimental design: 7-day-old rats (P7) were randomized according to sex and weight into four different treatment groups. Sham animals were not ligated and were only exposed to anesthesia for a short period of time (n = 15 animals per time point and condition). Common carotid ligation (CCL) was performed in the other groups, and animals were thereafter exposed to 90 min of hypoxia treatment (8% O_2_, 36 °C), resulting in moderate unilateral hypoxic-ischemic (HI) brain injury. After hypoxia (time point 1—TP1, n = 15 animals), HI animals were sacrificed immediately after HI. Following HI, the remaining ligated animals were treated with either normothermia treatment (NT) (37 °C) or hypothermia treatment (TH) (32 °C) for 5 h. Blood, spleen, bone marrow, and brains were isolated at different time points (immediately, 6 h, 24 h, and 48 h (n = 15 animals per time point and condition)) after HI. Animals from the last time point (seven days after HI) were sacrificed, and brain slices were stained with hematoxylin to measure the area loss after HI/NT (n = 10) or HI/TH treatment (n = 11). Made using BioRender.com.

Experiments were performed as previously described [46,94,97], following the ARRIVE guidelines and according to the Animal Protection Committee of the North Rhine-Westphalia State Environment Agency (LANUV), Germany. All studies were performed using 7-day-old (P7) Wistar rat pups of both sexes. Animals were kept at the central animal laboratory of the Deutsche Zentrum für Neurodegenerative Erkrankungen (DZNE) Bonn, Germany, with a 12:12 h dark/light cycle at an environmental temperature of 21 °C with food and water ad libitum. We performed randomization across litter, sex, and weight for all treatments before commencing experiments. All experiments and analysis were performed by observers blinded to the different treatments [52,98]. A total of 186 animals were used (90 females and 96 males) in different treatment groups and sacrificed at different time points (immediately after hypoxia, 6 h after hypoxia, 24 h after hypoxia, 48 h after hypoxia, and 7 days after hypoxia) (see Figure 6, made with BioRender.com (accessed on 15 August 2022)). Four groups were used: sham (n = 15 per time point), HI (n = 15 only for the time point immediately after HI), HI/NT (n = 15 per time point (6, 24, and 48 h after HI), and n = 10 for 7 days after HI), and HI/TH (n = 15 per time point (6, 24, and 48 h after HI)). Of the 15 animals, 10 were used for biochemistry and FACS analysis and 5 were used for immunohistochemistry. To check the efficiency of the treatment, we used 21 animals after 7 days of survival (n = 10 HI/NT and n = 11 HI/TH) for the analysis of brain tissue area loss. Temperature was monitored in “sentinel” rat pups not allocated to the different treatment groups during the experimental procedures, as previously described [46,94,97]. During all the experiments, the temperature of the rat pups was controlled by the sentinel pup via a rectal probe (IT-21, Physitemp Instruments, Clifton, NJ, USA), connected to a servo-controlled cooling machine and a cooling mat (CritiCool, MTRE, Yavne, Israel). The sentinel pup maintained the nesting temperature of the P7 rat pups [53] or treatment temperatures during the experiments (see below). The standardized HI Vannucci rat model was used, as previously described [46,94,97]. Briefly, the left common carotid artery was ligated and cut under general isoflurane anesthesia. Within 3 h, the pups were subjected to 8% O_2_ for 90 min at a rectal temperature (T_rectal_) of 36 °C, resulting in moderate HI brain injury [52,98]. Immediately after HI, pups were kept at a T_rectal_ of 37.0 °C, representing the normothermia treatment group, or at a T_rectal_ of 32.0 °C, representing the hypothermia treatment group, both for 5 h as previously described [10,52,98]. The pups were returned to their dams following the treatment period and sacrificed at different time points (Figure 6). For all pups at the first time point (immediately after hypoxia, TP1, see Figure 6), no normothermia or hypothermia treatment was performed. At the last time point (7 days after HI), the animals were sacrificed by transcardiac perfusion fixation with 10% neutral-buffered formalin, and the brains were kept in 10% neutral-buffered formalin until further processing. Coronal blocks were cut (3 mm sections) and embedded in paraffin. Tenmicrometer slices were cut from the two neighboring blocks representing the cortex, hippocampus, basal ganglia, and thalamus (distance to bregman -3.8 mm). The sections were stained with hematoxylin and eosin (HE) and the slices were scanned (Epson Perfection V750 Pro). The optical density and hemispheric area were analyzed using the ImageJ software. The ipsilateral side was compared to the contralateral side, and the area loss of the ligated side was calculated using the formula (1 − (area left/area right) × 100), as previously described [53,97,98].

### 4.2. Processing of Peripheral Blood, Bone Marrow, Spleen, and Brain Tissues for Flow Cytometry and Biochemistry Analysis

The sample processing was performed as follows: n = 10 animals per condition and time point. After sacrifice, the brains, spleens, blood, and bones were removed and kept on ice. The following procedure was performed for each tissue.

- Blood: Peripheral blood was collected in ethylenediaminetetraacetae (EDTA) 1.5 mL tubes (Eppendorf, Hamburg, Germany) and stored until further processing for a maximum of 30 min. Erythrocytes in blood samples were lysed by incubation with red blood cell lysis buffer (00-4333-57-Invitrogen-Darmstadt, Germany). The cells were washed three times and centrifuged at 300× *g* for 10 min at 4 °C. Finally, the isolated cells were resuspended in FACS buffer (2 mM EDTA (Sigma, Taufkirchen, Germany), 0.5% Bovine Serum Albumin (Sigma), and phosphate-buffered saline (PBS, Gibco, ThermoFisher, Neuss, Germany)). An aliquot of a single cell in suspension was used for flow cytometry analysis. The rest of the blood samples were centrifuged, and pellets were lysed with radioimmunoprecipitation assay buffer (RIPA (Millipore, Molsheim, France)) in the presence of phosphatase and protease inhibitors (Sigma) for 15 min on ice following centrifugation. Supernatants were collected and stored for subsequent biochemical analysis.
- Spleen: Spleens were dissected and homogenized through a 70 µm cell strainer (Falcon, Durham, USA) by washing with cold Hank’s Balanced Salt Solution (HBSS-Gibco-ThermoFischer, Schwerte, Germany). Erythrocytes in the spleen samples were lysed by incubation with red blood cell lysis buffer (00-4333-57-Invitrogen). Three steps of centrifugation at 300× *g* for 10 min at 4 °C and subsequent washing were performed. Finally, isolated cells were re-suspended in FACS buffer (2 mM EDTA (Sigma), 0.5% Bovine Serum Albumin (Sigma), and phosphate-buffered saline (PBS—Gibco—ThermoFisher)). An aliquot of single cells in suspension was used for flow cytometry analysis. The rest of the spleen samples were centrifuged, and pellets were lysed with radioimmunoprecipitation assay buffer (RIPA (Millipore)) in the presence of phosphatase and protease inhibitors (Sigma) for 15 min on ice following centrifugation. The supernatants were collected and stored for subsequent biochemical analysis.
- Bone Marrow: Bones were flushed with cold HBSS using a 29G needle and passed through a 70 µm cell strainer (Falcon) with continued washing with cold HBSS. Three centrifugation steps were performed at 300× *g* for 10 min at 4 °C and washing was performed for the cells from the bone marrow. Finally, isolated cells were re-suspended in FACS buffer (2 mM EDTA (Sigma), 0.5% Bovine Serum Albumin (Sigma), and phosphate-buffered saline (PBS—Gibco—ThermoFisher)). An aliquot of single cells in suspension was used for flow cytometry analysis. The rest of the bone marrow samples were centrifuged, and pellets were lysed for 15 min on ice with radioimmunoprecipitation assay buffer (RIPA (Millipore)) in the presence of phosphatase and protease inhibitors (Sigma) followed by centrifugation. Supernatants were collected and kept for posterior biochemistry analysis.
- Brain Samples: Separate ipsilateral brain hemispheres were homogenized using a glass homogenizer, passed through a 70 µm cell strainer (Falcon), and washed several times with cold HBSS. After centrifugation at 300× *g* for 10 min at 4 °C, the pellets from the homogenized brain samples were resuspended in 40% Percoll and added to a 15 mL Falcon tube with 80% Percoll. After 20 min of centrifugation at 1600× *g*, the myelin layer was removed and leukocytes in the interphase were collected following centrifugation at 300× *g* for 10 min. The pellets were then placed in FACS buffer and aliquots were collected for flow cytometry analysis. The rest of the brain samples were centrifuged, and pellets were lysed for 15 min on ice with radio-immunoprecipitation assay buffer (RIPA (Millipore)) in the presence of phosphatase and protease inhibitors (Sigma), followed by centrifugation. Supernatants were collected and kept for posterior biochemistry analysis.

The number of cells was counted using a Casy Cell Counter (OMNI Life Science, Bremen, Germany). A total of 5–10 million cells were used for bone marrow, spleen, and blood samples, while 1 million cells were used for brain samples.

### 4.3. Flow Cytometry

Flow cytometry staining was performed as previously described using specific antibody panels for myeloid cells in rat tissue, which included CD45 (clone OX-1), CD11b/c (clone OX-42), and the granulocyte marker RP-1 [99,100,101] comparable to Ly6g in mouse. These antibodies are equivalent to mouse tissue used in rats for myeloid lineages. CD45 (Ox-1) is a leukocyte-specific antigen expressed on all hematopoietic cells except erythrocytes. Granulocyte RP-1 is expressed on neutrophils during development and CD11b/c is expressed on all myeloid cells [99,100,102]. For surface staining, 10 × 10^6^ cells were transferred to a v-bottom 96-well plate and centrifuged at 300× *g* for 5 min. The cells were incubated with a combination of surface antibodies. The antibodies used are listed in Table 1. Briefly, the cells were resuspended in FACS buffer and stained with Zombie dye (BioLegend) in PBS. The cells were blocked with anti-CD32 (BD Pharming) to prevent Fc-mediated non-specific binding. Cells were then stained with antibodies in PBS at 4 °C for 30 min, washed, and fixed with 3% paraformaldehyde in PBS at room temperature for 15 min. Flow cytometry compensation was performed using UltraComp eBeads (Invitrogen) and flow cytometry was performed using a multiparameter FACSymphony A5 (BD Biosciences, Heidelberg, Germany). Thirty thousand events were recorded. The gate strategy was performed as previously described, and granulocytes were considered (Figure 1a) [99,100,102]. Briefly, we took advantage of the higher granularity of neutrophils, and the number of neutrophils was determined by quantifying the percentages of RP-1^+^ and CD11_b/c_^+^ cells from a CD45^+^ positive population [100,102]. Flow cytometry data were analyzed using FlowJo v.10.7.1 (National Institutes of Health, USA).

### 4.4. Biochemistry Analysis

The previously isolated samples were lysed in radioimmunoprecipitation (RIPA) buffer. Total protein concentration was measured using a bicinchoninic acid assay (BCA, Pierce^TM^ Thermo Fisher, Karlsruhe, Germany) following the manufacturer’s instructions. Immunoblotting was performed as previously described [97], with a few modifications. BIS-TRIS 12% gel was loaded with 50 µg total protein. SDS–polyacrylamide gel electrophoresis was performed in a morpholine ethanesulfonic acid buffer system (MOPS buffer, Thermo Fisher Scientific). The separated proteins were transferred onto a polyvinylidene difluoride membrane for 90 min at 110 V in transfer buffer containing 10% methanol. The membranes were blocked in protein-free buffer containing TBS with 0.05% (*v*/*v*) Tween 20 (MP Biomedical, Eschwege, Germany) for 1 h at room temperature, followed by incubation with the primary antibody incubation (Table 1) overnight at 4 °C. Secondary antibodies (Table 1), goat anti-mouse (IRDye 680 or IRDye 800) and goat-anti-rabbit (IRDye 800), were used to develop of the blots (LI-COR Biosciences, Bad Homburg, Germany) and imaged with an Odyssey infrared imaging system (LI-COR Biosciences). Optical density was determined using ImageJ and normalized to β-actin as a loading control. For those membranes which have antibodies with a different molecular weight between them, the membranes were reprobed, where the first antibody bound on the membrane was removed by washing twice with mild stripping buffer (200 mM Glycin (Sigma) pH 2.5 and 0.1% (*v*/*v*) Tween 20 (MP Biomedical)) for 15 min each time. The membrane was then washed with PBS 1× (Invitrogen) to recover the pH, followed by three washes with TBS with 0.05 (*v*/*v*) Tween 20 (MP Biomedical) (15 min each time). After washing, the membranes were blocked and reprobed with other antibodies.

### 4.5. Immunohistochemistry

Following transcardiac perfusion with phosphate-buffered saline (PBS) followed by 4% paraformaldehyde (Sigma-Aldrich), the brains were post-fixed in 4% paraformaldehyde overnight at 4 °C and embedded in paraffin. Immunohistochemistry was performed as previously described [103,104] using five animals per time point and group. We only used 24 h post-HI brain samples for immunohistochemistry, as some of the areas in the ipsilateral brain were severely injured, and technical preparation of the brain regions was not feasible. After deparaffinization, 10 µm coronal sections (−3.8 ± 0.7 mm from bregma) were rehydrated. Antigen retrieval was performed in pre-heated PBS 1× for 7 min following permeabilization with 0.1% Triton X-100 for 30 min at room temperature. After blocking with 20% normal goat serum in PBS 1× (Invitrogen, Frankfurt, Germany), slices were incubated with primary antibodies (Table 1) overnight at 4 °C, followed by incubation with the appropriate secondary antibody (Table 1) for 1 h at room temperature. Both primary and secondary antibodies were diluted in 0.7% carrageena solution with 0.02% NaN_3_ solution in PBS 1×. The sections were counterstained with 4,6-diamidino-2-phenylindole (DAPI) (Invitrogen). Immunohistochemistry was visualized by fluorescence microscopy AxioScan Z.1, using a 20× objective and confocal LSM900 (Zeiss, Oberkochem, Germany), using a 20× objective with a zoom of 2–3×. The images were analyzed using Zen 3.1 (Blue edition, Zeiss, Oberkochem, Germany) and ImageJ. We did not observe any differences in staining of the contralateral hemisphere. For quantification of the neutrophil-microglial distance (µm), scanned pictures with a 20× objective (AxioScan Z.1, Zeiss, Oberkochem, Germany) was performed as previously described with some modifications [69,105]. Certain criteria were followed, such as no overlap with neighboring cells and complete nucleus staining was taken into account. Cortical areas of the ipsilateral brain were divided into sections, and individual cells positive-stained for CIT-H3 and Iba-1 were selected. For the distance measurement, we used Zen 3.1 (Zeiss, Germany) software. The distance between the center of the mass of neutrophils and the nearest adjacent microglial cell was measured. A total of 300 cells derived from 5 animals per group (NT vs. TH) were measured.

### 4.6. Statistical Analysis

All analyses and data plots were made using GraphPad Prism 6 (GraphPad Software, La Jolla CA, USA). Two-way ANOVA or multiple *t*-tests (and nonparametric) with Tukey’s multiple comparison test were performed. To compare 2 groups, multiple *t*-tests with Holm–Ŝídák comparison were used. In all of our results, statistical significance was set at *p* < 0.05.

## Figures and Tables

**Table 1:** Antibodies list used for flow cytometry, immunohistochemistry and biochemistry.

Technique	Cat. N°	Antibody	Fluotochrome	Supplier	Clone	Dilution	Laser	Filter	Host
Flow Cytometry	740725	CD45	BV711	BDPharmingen	OX-1	1 in 200	Violet 355 nm	710/20	mouse
	201807	CD11 b/c	PE	BioLegend	OX-42	1 in 200	Yellow-Green 561 nm	596/15	mouse
	743056	RP-1	BV650	BDPharmingen	RP-1	1 in 200	Violet 355 nm	677/20	mouse
	77474	Zombie	UV	BioLegend	-	1 in 1000	Ultraviolet 350 nm	450/50	mouse
	202816	CD43	PE/Cy7	BioLegend	W3/13	1 in 200	Yellow-Green 561 nm	780/60	mouse
	550270	CD32	-	BDPharmingen	D34-485	1 in 200	-	-	mouse
Western Blot and IHC	AG-20B-0042	anti-Caspase-1	-	AdipoGen	p20	1 in 200	-	-	mouse
	ab5103	anti-Histone H3	-	abcam	-	1 in 500	-	-	rabbit
	66177-1-lg	anti-MPO	-	ProteinTech	4C11F6	1 in 500	-	-	mouse
	ab214185	anti-NLRP-3	-	abcam	-	1 in 500	-	-	rabbit
	A11008	Goat anti-rabbit Alexa Fluor 488	-	Invitrogen	-	1 in 400	-	-	rabbit
	A11005	Goat anti-mouse Alexa Fluor 594	-	Invitrogen	-	1 in 400	-	-	mouse
	35518	Goat anti-mouse DyLight 680	-	Invitrogen	-	1 in 2000	-	-	mouse
	926-32211	Goat anti-rabbit IRDye 800	-	Licor	-	1 in 2000	-	-	rabbit
IHC	17-9668-82	anti-Mo Ly-6G	APC	Invitrogen	1AB-Ly6g	1 in 500	-	-	mouse
	I32450	Isolectin GS-IB4	-	Invitrogen	-	1 in 200	-	-	rabbit
	ab5076	anti-IBA-1	-	Abcam	-	1 in 500	-	-	goat
	MAB377	NeuN	-	Millipore	A60	1 in 100	-	-	mouse

## Data Availability

Not applicable.

## Supplementary Materials

The following supporting information can be downloaded at https://www.mdpi.com/article/10.3390/ijms24043598/s1.

## References

[1] Dhillon S.K., Lear C.A., Galinsky R., Wassink G., Davidson J.O., Juul S., Robertson N.J., Gunn A.J., Bennet L. (2018). The fetus at the tipping point: Modifying the outcome of fetal asphyxia. J. Physiol..

[2] Ophelders D., Gussenhoven R., Klein L., Jellema R.K., Westerlaken R.J.J., Hutten M.C., Vermeulen J., Wassink G., Gunn A.J., Wolfs T.G. (2020). Preterm Brain Injury, Antenatal Triggers, and Therapeutics: Timing Is Key. Cells.

[3] Greco P., Nencini G., Piva I., Scioscia M., Volta C.A., Spadaro S., Neri M., Bonaccorsi G., Greco F., Cocco I. (2020). Pathophysiology of hypoxic–ischemic encephalopathy: A review of the past and a view on the future. Acta Neurol. Belg..

[4] Jacobs S.E., Berg M., Hunt R., Tarnow-Mordi W.O., Inder T.E., Davis P.G. (2013). Faculty Opinions recommendation of Cooling for newborns with hypoxic ischaemic encephalopathy. Cochrane Database Syst Rev..

[5] Nair J., Kumar V.H.S. (2018). Current and Emerging Therapies in the Management of Hypoxic Ischemic Encephalopathy in Neonates. Children.

[6] Aslam S., Strickland T., Molloy E.J. (2019). Neonatal Encephalopathy: Need for Recognition of Multiple Etiologies for Optimal Management. Front. Pediatr..

[7] Wang H., Liddell C.A., Coates M.M., Mooney M.D., Levitz C.E., Schumacher A.E., Apfel H., Iannarone M., Phillips B., Lofgren K.T. (2014). Global, regional, and national levels of neonatal, infant, and under-5 mortality during 1990–2013: A systematic analysis for the Global Burden of Disease Study. Lancet.

[8] Yenari M.A., Han H.S. (2012). Neuroprotective mechanisms of hypothermia in brain ischaemia. Nat. Rev. Neurosci..

[9] Sun Y.-J., Zhang Z.-Y., Fan B., Li G.-Y. (2019). Neuroprotection by Therapeutic Hypothermia. Front. Neurosci..

[10] Sabir H., Scull-Brown E., Liu X., Thoresen M. (2012). Immediate Hypothermia Is Not Neuroprotective After Severe Hypoxia-Ischemia and Is Deleterious When Delayed by 12 Hours in Neonatal Rats. Stroke.

[11] Hakobyan M., Dijkman K.P., Laroche S., Naulaers G., Rijken M., Steiner K., van Straaten H.L., Swarte R.M., ter Horst H.J., Zecic A. (2019). Outcome of Infants with Therapeutic Hypothermia after Perinatal Asphyxia and Early-Onset Sepsis. Neonatology.

[12] Danladi J., Sabir H. (2021). Perinatal Infection: A Major Contributor to Efficacy of Cooling in Newborns Following Birth Asphyxia. Int. J. Mol. Sci..

[13] Azevedo E.P., Guimaraes-Costa A.B., Torezani G.S., Braga C.A., Palhano F.L., Kelly J.W., Saraiva E.M., Foguel D. (2012). Amyloid fibrils trigger the release of neutrophil extracellular traps (NETs), causing fibril fragmentation by NET-associated elastase. J. Biol. Chem..

[14] Vaibhav K., Braun M., Alverson K., Khodadadi H., Kutiyanawalla A., Ward A., Banerjee C., Sparks T., Malik A., Rashid M.H. (2020). Neutrophil extracellular traps exacerbate neurological deficits after traumatic brain injury. Sci. Adv..

[15] Jorch S.K., Kubes P. (2017). An emerging role for neutrophil extracellular traps in noninfectious disease. Nat. Med..

[16] Cuartero M.I., Ballesteros I., Moraga A., Nombela F., Vivancos J., Hamilton J.A., Corbí Á.L., Lizasoain I., Moro M.A. (2013). N2 neutrophils, novel players in brain inflammation after stroke: Modulation by the PPARgamma agonist rosiglitazone. Stroke.

[17] Kim S.W., Davaanyam D., Seol S.I., Lee H.K., Lee H., Lee J.K. (2020). Adenosine Triphosphate Accumulated Following Cerebral Ischemia Induces Neutrophil Extracellular Trap Formation. Int. J. Mol. Sci..

[18] Kim S.W., Lee H., Lee H.K., Kim I.D., Lee J.K. (2019). Neutrophil extracellular trap induced by HMGB1 exacerbates damages in the ischemic brain. Acta Neuropathol. Commun..

[19] Otxoa-De-Amezaga A., Gallizioli M., Pedragosa J., Justicia C., Miró-Mur F., Salas-Perdomo A., Díaz-Marugan L., Gunzer M., Planas A.M. (2019). Location of Neutrophils in Different Compartments of the Damaged Mouse Brain After Severe Ischemia/Reperfusion. Stroke.

[20] Perez-De-Puig I., Miro-Mur F.A., Ferrer-Ferrer M., Gelpi E., Pedragosa J., Justicia C., Urra X., Chamorro A., Planas A.M. (2015). Neutrophil recruitment to the brain in mouse and human ischemic stroke. Acta Neuropathol..

[21] Lawrence S.M., Corriden R., Nizet V. (2017). Age-Appropriate Functions and Dysfunctions of the Neonatal Neutrophil. Front. Pediatr..

[22] Fernández-López D., Faustino J., Daneman R., Zhou L., Lee S.Y., Derugin N., Wendland M.F., Vexler Z.S. (2012). Blood-Brain Barrier Permeability Is Increased After Acute Adult Stroke But Not Neonatal Stroke in the Rat. J. Neurosci..

[23] Mulling K., Fischer A.J., Siakaeva E., Richter M., Bordbari S., Spyra I., Köster C., Hermann D.M., Gunzer M., Felderhoff-Müser U. (2020). Neutrophil dynamics, plasticity and function in acute neurodegeneration following neonatal hypoxia-ischemia. Brain Behav. Immun..

[24] Shrivastava K., Chertoff M., Llovera G., Recasens M., Acarin L. (2012). Short and Long-Term Analysis and Comparison of Neurodegeneration and Inflammatory Cell Response in the Ipsilateral and Contralateral Hemisphere of the Neonatal Mouse Brain after Hypoxia/Ischemia. Neurol. Res. Int..

[25] Grether J.K. (1997). Maternal infection and cerebral palsy in infants of normal birth weight. JAMA J. Am. Med. Assoc..

[26] Morkos A.A., Hopper A.O., Deming D.D., Yellon S.M., Wycliffe N., Ashwal S., Sowers L.C., Peverini R.L., Angeles D.M. (2007). Elevated total peripheral leukocyte count may identify risk for neurological disability in asphyxiated term neonates. J. Perinatol..

[27] Povroznik J.M., Engler-Chiurazzi E.B., Nanavati T., Pergami P. (2018). Absolute lymphocyte and neutrophil counts in neonatal ischemic brain injury. SAGE Open Med..

[28] Yost C.C., Schwertz H., Cody M.J., Wallace J.A., Campbell R.A., Vieira-de-Abreu A., Araujo C.V., Schubert S., Harris E.S., Rowley J.W. (2016). Neonatal NET-inhibitory factor and related peptides inhibit neutrophil extracellular trap formation. J. Clin. Investig..

[29] Doycheva D.M., Hadley T., Li L., Applegate R.L., Zhang J.H., Tang J. (2014). Anti-neutrophil antibody enhances the neuroprotective effects of G-CSF by decreasing number of neutrophils in hypoxic ischemic neonatal rat model. Neurobiol. Dis..

[30] Palmer C., Roberts R.L., Young P.I. (2004). Timing of neutrophil depletion influences long-term neuroprotection in neonatal rat hypoxic-ischemic brain injury. Pediatr. Res..

[31] Mallard C., Tremblay M.E., Vexler Z.S. (2018). Microglia and Neonatal Brain Injury. Neuroscience.

[32] Herz J., Bendix I., Felderhoff-Muser U. (2022). Peripheral immune cells and perinatal brain injury: A double-edged sword?. Pediatr. Res..

[33] Chen G.Y., Nunez G. (2010). Sterile inflammation: Sensing and reacting to damage. Nat. Rev. Immunol..

[34] Li Q., Barres B.A. (2018). Microglia and macrophages in brain homeostasis and disease. Nat. Rev. Immunol..

[35] Saijo K., Glass C.K. (2011). Microglial cell origin and phenotypes in health and disease. Nat. Rev. Immunol..

[36] Yang Q.Q., Zhou J.W. (2019). Neuroinflammation in the central nervous system: Symphony of glial cells. Glia.

[37] Salah M.M., Abdelmawla M.A., Eid S.R., Hasanin R.M., Mostafa E.A., Abdelhameed M.W. (2019). Role of Matrix Metalloproteinase-9 in Neonatal Hypoxic-Ischemic Encephalopathy. Open Access Maced J. Med. Sci..

[38] Liu F., McCullough L.D. (2013). Inflammatory responses in hypoxic ischemic encephalopathy. Acta Pharmacol. Sin..

[39] Li B., Concepcion K., Meng X., Zhang L. (2017). Brain-immune interactions in perinatal hypoxic-ischemic brain injury. Prog. Neurobiol..

[40] Strecker J.K., Schmidt A., Schabitz W.R., Minnerup J. (2017). Neutrophil granulocytes in cerebral ischemia—Evolution from killers to key players. Neurochem. Int..

[41] Nazmi A., Albertsson A.-M., Rocha-Ferreira E., Zhang X., Vontell R., Zelco A., Rutherford M., Zhu C., Nilsson G., Mallard C. (2018). Lymphocytes Contribute to the Pathophysiology of Neonatal Brain Injury. Front. Neurol..

[42] Daniel C., Leppkes M., Muñoz L.E., Schley G., Schett G., Herrmann M. (2019). Extracellular DNA traps in inflammation, injury and healing. Nat. Rev. Nephrol..

[43] Chen Y., Zhang H., Hu X., Cai W., Ni W., Zhou K. (2022). Role of NETosis in Central Nervous System Injury. Oxidative Med. Cell. Longev..

[44] Pérez-Figueroa E., Álvarez-Carrasco P., Ortega E., Maldonado-Bernal C. (2021). Neutrophils: Many Ways to Die. Front. Immunol..

[45] Ystgaard M.B., Sejersted Y., Loberg E.M., Lien E., Yndestad A., Saugstad O.D. (2015). Early Upregulation of NLRP3 in the Brain of Neonatal Mice Exposed to Hypoxia-Ischemia: No Early Neuroprotective Effects of NLRP3 Deficiency. Neonatology.

[46] Bernis M.E., Schleehuber Y., Zweyer M., Maes E., Felderhoff-Müser U., Picard D., Sabir H. (2022). Temporal Characterization of Microglia-Associated Pro- and Anti-Inflammatory Genes in a Neonatal Inflammation-Sensitized Hypoxic-Ischemic Brain Injury Model. Oxidative Med. Cell. Longev..

[47] Grebe A., Hoss F., Latz E. (2018). NLRP3 Inflammasome and the IL-1 Pathway in Atherosclerosis. Circ. Res..

[48] Johnson J.L., Ramadass M., Haimovich A., McGeough M.D., Zhang J., Hoffman H.M., Catz S.D. (2017). Increased Neutrophil Secretion Induced by NLRP3 Mutation Links the Inflammasome to Azurophilic Granule Exocytosis. Front. Cell. Infect. Microbiol..

[49] Münzer P., Negro R., Fukui S., di Meglio L., Aymonnier K., Chu L., Cherpokova D., Gutch S., Sorvillo N., Shi L. (2021). NLRP3 Inflammasome Assembly in Neutrophils Is Supported by PAD4 and Promotes NETosis Under Sterile Conditions. Front. Immunol..

[50] Bakele M., Joos M., Burdi S., Allgaier N., Pöschel S., Fehrenbacher B., Schaller M., Marcos V., Kümmerle-Deschner J., Rieber N. (2014). Localization and Functionality of the Inflammasome in Neutrophils. J. Biol. Chem..

[51] Kelley N., Jeltema D., Duan Y., He Y. (2019). The NLRP3 Inflammasome: An Overview of Mechanisms of Activation and Regulation. Int. J. Mol. Sci..

[52] Osredkar D., Sabir H., Falck M., Wood T., Maes E., Flatebø T., Puchades M., Thoresen M. (2015). Hypothermia Does Not Reverse Cellular Responses Caused by Lipopolysaccharide in Neonatal Hypoxic-Ischaemic Brain Injury. Dev. Neurosci..

[53] Wood T., Osredkar D., Puchades M., Maes E., Falck M., Flatebø T., Walløe L., Sabir H., Thoresen M. (2016). Treatment temperature and insult severity influence the neuroprotective effects of therapeutic hypothermia. Sci. Rep..

[54] Wu X., Zeng H., Cai L., Chen G. (2021). Role of the Extracellular Traps in Central Nervous System. Front. Immunol..

[55] Banerjee S.K., Chatterjee A., Gupta S., Nagar A. (2022). Activation and Regulation of NLRP3 by Sterile and Infectious Insults. Front. Immunol..

[56] Alishahi M., Farzaneh M., Ghaedrahmati F., Nejabatdoust A., Sarkaki A., Khoshnam S.E. (2019). NLRP3 inflammasome in ischemic stroke: As possible therapeutic target. Int. J. Stroke.

[57] Kang L., Yu H., Yang X., Zhu Y., Bai X., Wang R., Cao Y., Xu H., Luo H., Lu L. (2020). Neutrophil extracellular traps released by neutrophils impair revascularization and vascular remodeling after stroke. Nat. Commun..

[58] Bona E., Hagberg H., Løberg E.M., Bågenholm R., Thoresen M. (1998). Protective Effects of Moderate Hypothermia after Neonatal Hypoxia-Ischemia: Short- and Long-Term Outcome. Pediatr. Res..

[59] Iwata O., Iwata S., Thornton J., De Vita E., Bainbridge A., Herbert L., Scaravilli F., Peebles D., Wyatt J.S., Cady E.B. (2007). “Therapeutic time window” duration decreases with increasing severity of cerebral hypoxia–ischaemia under normothermia and delayed hypothermia in newborn piglets. Brain Res..

[60] Thoresen M., Tooley J., Liu X., Jary S., Fleming P., Luyt K., Jain A., Cairns P., Harding D., Sabir H. (2013). Time Is Brain: Starting Therapeutic Hypothermia within Three Hours after Birth Improves Motor Outcome in Asphyxiated Newborns. Neonatology.

[61] Yenari M.A., Han H.S. (2013). Influence of therapeutic hypothermia on regeneration after cerebral ischemia. Front. Neurol. Neurosci..

[62] Peretti D., Bastide A., Radford H., Verity N., Molloy C., Martin M.G., Moreno J.A., Steinert J.R., Smith T., Dinsdale D. (2015). RBM3 mediates structural plasticity and protective effects of cooling in neurodegeneration. Nature.

[63] Fleiss B., Van Steenwinckel J., Bokobza C., Shearer I.K., Ross-Munro E., Gressens P. (2021). Microglia-Mediated Neurodegeneration in Perinatal Brain Injuries. Biomolecules.

[64] Gong T., Liu L., Jiang W., Zhou R. (2020). DAMP-sensing receptors in sterile inflammation and inflammatory diseases. Nat. Rev. Immunol..

[65] Yao H.W., Kuan C.Y. (2020). Early neutrophil infiltration is critical for inflammation-sensitized hypoxic-ischemic brain injury in newborns. J. Cereb. Blood Flow Metab..

[66] Neumann J., Henneberg S., Von Kenne S., Nolte N., Müller A.J., Schraven B., Görtler M.W., Reymann K.G., Gunzer M., Riek-Burchardt M. (2018). Beware the intruder: Real time observation of infiltrated neutrophils and neutrophil—Microglia interaction during stroke in vivo. PLoS ONE.

[67] Kratzer I., Chip S., Vexler Z.S. (2014). Barrier mechanisms in neonatal stroke. Front. Neurosci..

[68] Hijioka M., Futokoro R., Ohto-Nakanishi T., Nakanishi H., Katsuki H., Kitamura Y. (2020). Microglia-released leukotriene B4 promotes neutrophil infiltration and microglial activation following intracerebral hemorrhage. Int. Immunopharmacol..

[69] Neumann J., Riek-Burchardt M., Herz J., Doeppner T.R., König R., Hütten H., Etemire E., Männ L., Klingberg A., Fischer T. (2015). Very-late-antigen-4 (VLA-4)-mediated brain invasion by neutrophils leads to interactions with microglia, increased ischemic injury and impaired behavior in experimental stroke. Acta Neuropathol..

[70] Enzmann G., Mysiorek C., Gorina R., Cheng Y.-J., Ghavampour S., Hannocks M.-J., Prinz V., Dirnagl U., Endres M., Prinz M. (2012). The neurovascular unit as a selective barrier to polymorphonuclear granulocyte (PMN) infiltration into the brain after ischemic injury. Acta Neuropathol..

[71] Weisenburger-Lile D., Dong Y., Yger M., Weisenburger G., Polara G.F., Chaigneau T. (2019). Harmful neutrophil subsets in patients with ischemic stroke: Association with disease severity. Neurol Neuroimmunol Neuroinflamm..

[72] Kurt A., Zenciroğlu A., Akduman H. (2022). The impact of therapeutic hypothermia on peripheral blood cell in newborns with hypoxic ischemic encephalopathy. Braz. J. Pharm. Sci..

[73] O’Dea M.I., Kelly L., McKenna E., Melo A.M., Ni Bhroin M., Hurley T., Byrne A.T., Colleran G., Vavasseur C., El-Khuffash A. (2021). Dysregulated Monocyte and Neutrophil Functional Phenotype in Infants With Neonatal Encephalopathy Requiring Therapeutic Hypothermia. Front. Pediatr..

[74] Shelley M. (2020). Lawrence RCaVN. How Neutrophils Meet Their End. Cell.

[75] Hidalgo A., Chilvers E.R., Summers C., Koenderman L. (2019). The Neutrophil Life Cycle. Trends Immunol..

[76] Zenaro E., Pietronigro E., Della Bianca V., Piacentino G., Marongiu L., Budui S., Turano E., Rossi B., Angiari S., Dusi S. (2015). Neutrophils promote Alzheimer’s disease-like pathology and cognitive decline via LFA-1 integrin. Nat. Med..

[77] Zeng H., Fu X., Cai J., Sun C., Yu M., Peng Y., Zhuang J., Chen J., Chen H., Yu Q. (2022). Neutrophil Extracellular Traps may be a Potential Target for Treating Early Brain Injury in Subarachnoid Hemorrhage. Transl. Stroke Res..

[78] Jamali E., Abbasi M., Tayer A.H., Monfared A.A., Tandel P., Tamaddon G., Kazerooni E.S., Rakhshandehroo S., Ranjbaran R. (2022). The significance of surface neutrophilic MPO expression level in NETosis and NETosis-associated coagulopathies in covid-19 infected patients. Blood Cells, Mol. Dis..

[79] Baj N.T. (1994). Comparison of Properties of Membrane Bound Versus Soluble Forms of Human Leukocytic Elastase and Cathepsin, G. Biol. Chem..

[80] Sabbatini M., Magnelli V., Renò F. (2021). NETosis in Wound Healing: When Enough Is Enough. Cells.

[81] Hahn J., Schauer C., Czegley C., Kling L., Petru L., Schmid B., Weidner D., Reinwald C., Biermann M.H.C., Blunder S. (2019). Aggregated neutrophil extracellular traps resolve inflammation by proteolysis of cytokines and chemokines and protection from antiproteases. FASEB J..

[82] Liang X., Liu L., Wang Y., Guo H., Fan H., Zhang C., Hou L., Liu Z. (2020). Autophagy-driven NETosis is a double-edged sword—Review. Biomed. Pharmacother..

[83] Rosales C. (2018). Neutrophil: A Cell with Many Roles in Inflammation or Several Cell Types?. Front. Physiol..

[84] Fukushima K., Nabeshima H., Kida H. (2021). Revealing the diversity of neutrophil functions and subsets. Cell. Mol. Immunol..

[85] Cahilog Z., Zhao H., Wu L., Alam A., Eguchi S., Weng H., Ma D. (2020). The Role of Neutrophil NETosis in Organ Injury: Novel Inflammatory Cell Death Mechanisms. Inflammation.

[86] Allen C., Thornton P., Denes A., McColl B.W., Pierozynski A., Monestier M., Pinteaux E., Rothwell N.J., Allan S.M. (2012). Neutrophil Cerebrovascular Transmigration Triggers Rapid Neurotoxicity through Release of Proteases Associated with Decondensed DNA. J. Immunol..

[87] Denorme F., Portier I., Rustad J.L., Cody M.J., de Araujo C.V., Hoki C., Alexander M.D., Grandhi R., Dyer M.R., Neal M.D. (2022). Neutrophil extracellular traps regulate ischemic stroke brain injury. J. Clin. Investig..

[88] Mottahedin A., Blondel S., Ek J., Leverin A.-L., Svedin P., Hagberg H., Mallard C., Ghersi-Egea J.-F., Strazielle N. (2020). N-acetylcysteine inhibits bacterial lipopeptide-mediated neutrophil transmigration through the choroid plexus in the developing brain. Acta Neuropathol. Commun..

[89] Lange S., Rocha-Ferreira E., Thei L., Mawjee P., Bennett K., Thompson P.R., Subramanian V., Nicholas A.P., Peebles D., Hristova M. (2014). Peptidylarginine deiminases: Novel drug targets for prevention of neuronal damage following hypoxic ischemic insult (HI) in neonates. J. Neurochem..

[90] Lewis H.D., Liddle J., Coote J.E., Atkinson S.J., Barker M.D., Bax B.D., Bicker K.L., Bingham R.P., Campbell M., Chen Y.H. (2015). Inhibition of PAD4 activity is sufficient to disrupt mouse and human NET formation. Nat. Chem. Biol..

[91] Tomura S., de Rivero Vaccari J.P., Keane R.W., Bramlett H.M., Dietrich W.D. (2012). Effects of therapeutic hypothermia on inflammasome signaling after traumatic brain injury. J. Cereb. Blood Flow. Metab..

[92] Gao R., Zhao H., Wang X., Tang B., Cai Y., Zhang X., Zong H., Li Y., Wang Y. (2021). Mild Hypothermia Therapy Lowers the Inflammatory Level and Apoptosis Rate of Myocardial Cells of Rats with Myocardial Ischemia-Reperfusion Injury via the NLRP3 Inflammasome Pathway. Comput. Math. Methods Med..

[93] Zhang J., Lu Y., Yu P., Li Z., Liu Y., Zhang J., Yu S. (2022). Therapeutic hypothermia alleviates myocardial ischaemia-reperfusion injury by inhibiting inflammation and fibrosis via the mediation of the SIRT3/NLRP3 signalling pathway. J. Cell Mol. Med..

[94] Serdar M., Kempe K., Rizazad M., Herz J., Bendix I., Felderhoff-Müser U., Sabir H. (2019). Early Pro-inflammatory Microglia Activation After Inflammation-Sensitized Hypoxic-Ischemic Brain Injury in Neonatal Rats. Front. Cell. Neurosci..

[95] Cassel S.L., Sutterwala F.S. (2010). Sterile inflammatory responses mediated by the NLRP3 inflammasome. Eur. J. Immunol..

[96] McKee C.M., Coll R.C. (2020). NLRP3 inflammasome priming: A riddle wrapped in a mystery inside an enigma. J. Leukoc. Biol..

[97] Serdar M., Kempe K., Herrmann R., Picard D., Remke M., Herz J., Bendix I., Felderhoff-Müser U., Sabir H. (2020). Involvement of CXCL1/CXCR2 During Microglia Activation Following Inflammation-Sensitized Hypoxic-Ischemic Brain Injury in Neonatal Rats. Front. Neurol..

[98] Osredkar D., Thoresen M., Maes E., Flatebo T., Elstad M., Sabir H. (2013). Hypothermia is not Neuroprotective after Infection-Sensitized Neonatal Hypoxic-Ischemic Brain Injury. Resuscitation.

[99] Barnett-Vanes A., Sharrock A., Birrell M.A., Rankin S. (2016). A Single 9-Colour Flow Cytometric Method to Characterise Major Leukocyte Populations in the Rat: Validation in a Model of LPS-Induced Pulmonary Inflammation. PLoS ONE.

[100] Francis W.R., Ireland R.E., Spear A.M., Jenner D., Watts S.A., Kirkman E., Pallister I. (2019). Flow Cytometric Analysis of Hematopoietic Populations in Rat Bone Marrow. Impact of Trauma and Hemorrhagic Shock. Cytometry A.

[101] Manglani M., Gossa S., McGavern D.B. (2018). Leukocyte Isolation from Brain, Spinal Cord, and Meninges for Flow Cytometric Analysis. Curr. Protoc. Immunol..

[102] Grau V., Scriba A., Stehling O., Steiniger B. (2000). Monocytes in the Rat. Immunobiology.

[103] Reinboth B.S., Köster C., Abberger H., Prager S., Bendix I., Felderhoff-Müser U., Herz J. (2016). Endogenous hypothermic response to hypoxia reduces brain injury: Implications for modeling hypoxic-ischemic encephalopathy and therapeutic hypothermia in neonatal mice. Exp. Neurol..

[104] Serdar M., Herz J., Kempe K., Lumpe K., Reinboth B.S., Sizonenko S.V., Hou X., Herrmann R., Hadamitzky M., Heumann R. (2016). Fingolimod protects against neonatal white matter damage and long-term cognitive deficits caused by hyperoxia. Brain Behav. Immun..

[105] Herz J., Sabellek P., Lane T.E., Gunzer M., Hermann D.M., Doeppner T.R. (2015). Role of Neutrophils in Exacerbation of Brain Injury After Focal Cerebral Ischemia in Hyperlipidemic Mice. Stroke.

