# Supplementary material for: Neutrophil Extracellular Traps Release following Hypoxic-Ischemic Brain Injury in Newborn Rats Treated with Therapeutic Hypothermia
Source: Int J Mol Sci. 2023 Feb 10;24(4):3598. doi: 10.3390/ijms24043598 (PMC9966013; doi:10.3390/ijms24043598)
Supplement: Supplementary file 1 [file ijms-24-03598-s001.zip › ijms-2130085-supplementary.pdf]

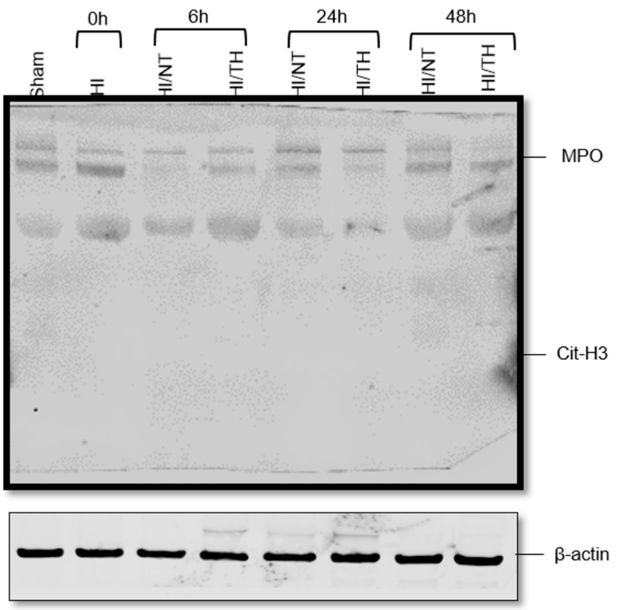

Supplementary Figure S1. Representative Western blotting of blood samples at different time points following different treatments. MPO was added to demonstrate the presence of neutrophil markers in isolated blood. Cit-H3 did not show any positive band. β-actin was used as a loading control.
